# Evidencing Protective and Risk Factors for Harmful Alcohol Drinking in Adolescence: A Prospective Analysis of Sport-Participation and Scholastic-Achievement in Older Adolescents from Croatia

**DOI:** 10.3390/ijerph15050986

**Published:** 2018-05-14

**Authors:** Sime Devcic, Damir Sekulic, Divo Ban, Zvonimir Kutlesa, Jelena Rodek, Dorica Sajber

**Affiliations:** 1 Special Hospital Biograd n/m, Biograd 23120, Croatia; cicved@gmail.com; 2 Faculty of Medicine, University of Mostar, Mostar 88000, Bosnia and Herzegovina; 3 Faculty of Kinesiology, University of Split, Split 21000, Croatia; djivo.ban@du.t-com.hr (D.B.); jrodek@kifst.hr (J.R.); 4 University of Dubrovnik, Dubrovnik 20000, Croatia; 5 Clinical Hospital Split, Split 21000, Croatia; zvonimir.kutlesa@gmail.com; 6 Faculty of Sport, University of Ljubljana, Ljubljana 1000, Slovenia; dorica.sajber@fsp.uni-lj.si

**Keywords:** substance abuse, educational achievement, physical exercise, puberty, relationships

## Abstract

*Background*: The prevalence of alcohol drinking (AD) in Croatian adolescents is alarming, but there is an evident lack of prospective analyses of the protective/risk factors of AD. This study aimed to prospectively investigate the relationships between scholastic and sport factors and harmful alcohol drinking (HD) in older adolescents. *Methods*: The participants (n = 644, 53.7% females) were 16 years of age at study baseline and were tested at baseline and again 20 months later (follow-up). The predictors included four variables of scholastic achievement and four factors evidencing involvement in sport. Criterion was AD observed on the Alcohol Use Disorders Identification Test (AUDIT), and results were later categorized into harmful drinking (HD) and non-harmful drinking (NHD). The HD at baseline, HD at follow-up and HD initiation during the study course were observed as criteria in logistic regression analyses, which were additionally controlled for confounders (age, gender, socioeconomic status, and conflict with parents). *Results:* With 22% and 29% adolescents who reported HD at baseline and follow-up, respectively, the prevalence of HD remains among the highest in Europe. Scholastic failure was systematically related to HD at baseline and follow-up, but scholastic variables did not predict HD initiation during the course of the study. The higher odds for HD at baseline were evidenced for current and former team sport athletes. Those who quit individual sport were more likely to engage in HD at follow-up. Longer involvement in sport (OR: 2.10, 95% CI: 1.18–3.72), higher sport result (OR: 3.15, 95% CI: 1.19–8.34), and quitting individual sport (OR: 13.13, 95% CI: 2.67–64.62) were predictive of HD initiation. *Conclusions*: The results indicated specific associations between sport factors with HD initiation, which is understandable knowing the high stress placed on young athletes in this period of life, mainly because of the forthcoming selection between junior (amateur) and senior (professional) level. The results did not allow interpretation of the cause-effect relationship between scholastic failure and HD in the studied period.

## 1. Introduction

Apart from being a serious health-threatening behavior, alcohol is directly and indirectly connected to numerous negative social consequences, such as risky sexual behavior, family violence, physical abuse, and traffic accidents [1,2,3,4,5,6,7,8,9]. Drinking alcohol is a particularly important problem in adolescence. First, young brains are very vulnerable because the brain structures are still developing during puberty, and alcohol can damage some parts of the brain, consequently affecting the learning abilities and behavior of young people [10,11]. Additionally, alcohol consumption is known to be related to aggressive behaviors, which is a clear link toward criminal behavior, which implies serious legal consequences [12]. Further, in comparison to older persons, adolescents run a greater risk of alcohol poisoning when they consume large quantities of alcohol in a short period of time [13]. Studies have frequently explored factors for being potentially related to alcohol consumption in adolescents [14,15,16,17,18]. 

It is not surprising that studies frequently examined the associations that may exist between educational achievement (scholastic factors) and alcohol consumption in adolescents [16,19,20,21]. In short, alcohol consumption was systematically correlated to poor performance in school observed on a basis of grade-point-average (GPA), absence from school, number of schooling years, etc. [22,23,24]. However, while physiological explanation of causality between alcohol drinking and poor scholastic achievement seems sound (i.e., alcohol negatively influences learning capacities), the cause-effect relationship between alcohol and achievement in school can be explained vice-versa by certain socio-cultural contexts of alcohol consumption in adolescence. Specifically, teenagers who perform poorly in school are often in out-of-school and in non-controlled social environments, where they are more likely to start drinking alcohol excessively [25]. 

The socio-cultural context of alcohol drinking behavior in adolescence is particularly evident in discussions of associations between sport participation and consumption of alcohol [24,25]. It has been suggested that sport participation may prevent problem behavior during adolescence, and this contention also concurs with etiological theories [26,27]. However, studies more often did not than did confirm protective effects of participation in sport against alcohol consumption in youth [25,28,29,30]. In brief, since some investigators reported sports as being protective against drinking alcohol [31], a great deal of studies highlighted sports participation as a factor contributing to increased risk for alcohol consumption and binge drinking [25,28,29,30]. Again, differences in observed associations are explained regarding differences in the type of sport (i.e., differential influence of individual- and team-sport participation) [25], sex-specific factors [32], the level of sport participation (i.e., sport achievement) [23], social circumstances (i.e., parental influence, socioeconomic status) [25], and the role of teammates, friendship and popularity [30]. However, most of the studies that examined the associations were cross-sectional, and therefore, causality between sport participation and alcohol drinking in adolescence remains unknown. 

Cross-sectional studies in south-eastern Europe, including Croatia reported that approximately 30% of adolescents drink alcohol at harmful level (based on the Alcohol Use Disorders Identification Test (AUDIT), a scale proposed by the World Health Organization), which is alarming prevalence in comparison to other European countries [22,23,32,33,34]. Because of such high alcohol consumption statistics, several studies from the region have investigated the associations between drinking alcohol and sport participation in older adolescents. In Croatian adolescents, sport participation factors were poorly related to drinking alcohol in males, but sport participation was evidently higher in girls who had higher AUDIT scores [32]; similar results were reported in studies on adolescents from Bosnia and Herzegovina, with a higher occurrence of harmful drinking in girls who achieved better success in sports [24,35]. Prior sport participation was identified as a risk factor for the higher occurrence of harmful alcohol consumption in Kosovar adolescents, but there was no difference in the prevalence of harmful drinking behaviors between adolescents who were currently involved in sports and those that were never involved in sports [23]. 

While previous studies done in the region were cross-sectional [22,23,32], the clear cause-effect relationship between scholastic- and sport-factors, and alcohol drinking prevalence is not known. Therefore [23,24,32,35] this prospective study aimed to establish the possible influence of the sport, scholastic and certain socio-demographic factors on the initiation of harmful alcohol drinking in older adolescents from Croatia. 

## 2. Materials and Methods 

### 2.1. Participants and Testing

**Figure 1:**
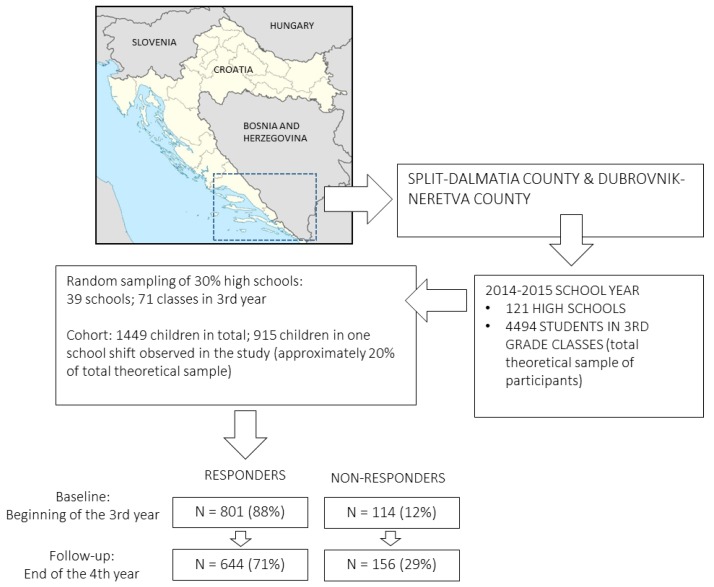
Study location, testing waves, participant- and drop-out-rates.

In this study, we observed 16-to-18-year-old adolescents (n = 644, 54% females). The sample comprised adolescents from Dubrovnik-Neretva and Split-Dalmatia regions, both located in the south of Croatia, on the coast of the Adriatic Sea (Figure 1). While the idea of the study was to evidence scholastic and sport factors potentially related to alcohol drinking, it was necessary to obtain a sample of participants in the regions with similar heritage and culture (Mediterranean culture and tradition), especially for the most popular types of sport (i.e., water sports in our sample). Originally, the sample included 915 participants, but only those who were observed over two testing waves (see later text for details) were included in the study. 

Sampling was based on a multi-stage simple random sampling method. At the first stage, all high schools in the observed cities were sorted into three groups according to number of pupils. In the second phase, we randomly selected 30% of schools from each group (39 schools). Next, one-school shift in the chosen schools was randomly selected. This process altogether resulted in a cohort of 915 children. The study was anonymous, and no personal data were gathered, but to follow responses over testing waves, participants were asked to use a self-selected confidential code (i.e., last three digits of their e-mail password). Ethical approval was obtained from the Ethical Board of the corresponding author’s institution (Ethical Approval No: 2181-205-02-05-14-005). During the first week of the school year, at the regular school meeting, the study design and aims of the investigation were presented to parents/guardians of the children, who gave written consent for participation in the study. 

The testing was done over two waves: baseline (beginning of the 3rd year of high school; participants were 16 years old on average) and follow-up (approximately 20 months later). Testing was organized during school hours and took approximately 15 minutes. When testing was done, participants sealed the questionnaire form and put it in a closed box. Baseline and follow-up testing were done only once, and adolescents who were not at school on a testing day were not included in the study (please see Figure 1 for study design, response rates and drop-outs). 

### 2.2. Variables

The variables in this study included data on questions about predictors (sport factors and scholastic -educational factors), criteria (alcohol drinking behavior) and covariates (parental conflict, socioeconomic status, age and sex). 

Surveys were performed using an extensive self-administered questionnaire, which was previously applied and found to be valid in a similar sample of subjects in surrounding countries [22,25,32]. In general, all questions except the question on participant age were structured as multiple choice. 

Sport factors were evaluated by four questions: (i) participation in competitive individual sports; (ii) participation in competitive team sports (both (i) and (ii) are answered on a three-point scale: never been involved, quit, I’m currently involved); (iii) competitive result achieved in sport (never involved/competed, local competitions, national-level competition, international-level competition); and (iv) experience in sport (never been involved in sport, <1 year, 2–5 years, >5 years). 

The scholastic achievement (educational achievement) included the following questions: (i) GPA (answered on a five-point scale ranging from “excellent” to “failed”); (ii) behavioral grade (three-point scale: exceptional–average–poor); (iii) number of hours absent from school (<10, 10–20, 21–40, >40 hours); and (iv) number of unexcused hours absent from school (none, 1–5, 6–10, 11–20, >20). 

Alcohol consumption was measured using the AUDIT questionnaire, proposed by the World Health Organization (WHO) [36]. The AUDIT contains 10 items with scores ranging from 0 to 4 resulting in a scale ranging from a theoretical minimum (0) to a maximum (40). Participants were ultimately divided into two groups according to overall AUDIT score; one group contained participants involved in “harmful drinking” (HD), and the other comprised those who consumed alcohol at a “non-harmful level” (non-harmful-drinking—NHD). Although the literature suggests two approaches for dividing scores into HD and NHD (i.e., by using the scores of 8 and 11 as a “cut-of-point”), for a meaningful comparison with previous studies from the territory, we have used a total score of 11 as a cut-off score for HD [22,25,35]. 

Covariates (i.e., confounding factors) included participants’ age, sex, self-reported socioeconomic status (SES (socioeconomic status): under average–average–above average), and self-perceived level of conflict with parents (four-point scale from “no conflict at all” to “frequently”). 

The analysis of attrition bias showed no significant differences in the initial harmful drinking status between the participants who dropped out and those who remained in the study (Chi-square: 0.01, *p* = 0.91). The intracluster correlation coefficient calculated for baseline harmful drinking prevalence, with schools observed as clusters, was 0.07, indicating appropriate within-cluster (i.e., within-school) variance [37]. 

### 2.3. Statistics

Depending on the parametric/nonparametric nature of the variables (which was checked by the Kolmogorov-Smirnov test), descriptive statistics included means and standard deviations (for parametric) and frequencies and percentages (for nonparametric variables). The differences between the groups (HD vs. NHD) were established by chi-square test, or the Mann-Whitney test. To define correlates of HD at baseline (HD-baseline), HD at follow-up (HD-follow-up), and initiation of HD during the study (HD-initiation) binary logistic regression was calculated, and odds ratio (OR) with a corresponding 95% confidence interval (CI) were presented. Crude regression models were additionally controlled for age, sex, SES and parental conflict as confounding variables (covariates). 

## 3. Results

Overall, the HD is identified in 22.3% of adolescents at the baseline, and in 28.6% at the end of the high-school, with higher odds for HD in males (OR: 2.00, 95% CI: 1.3–2.9; OR: 1.92, 95% CI: 1.4–2.8 for baseline and follow-up, respectively). 

At baseline and follow-up, non-users were less absent from school (MW: 2.72 (*p* < 0.01), 4.56 (0.01)), had less unexcused absences (3.65 (0.01), 5.20 (0.01)) (4.49 (0.01), 2.98 (0.01), for baseline and follow-up, respectively). Also, HD had lower GPA at baseline (3.26 (0.01)), and lower behavioral grade at follow-up (3.14 (0.01)) (Table 1).

Those who were identified as HD at baseline were more likely to be involved in team sports at both testing waves (Chi square: 20.88, *p* < 0.01, 9.18, 0.01; for baseline and follow-up, respectively), and individual sports at follow up (8.10, 0.02) (Table 2). 

Those adolescents were identified as HD reported lower level of conflict with their parents (responsible adults) at baseline (MW: 4.17, *p* < 0.01) (Table 3).

Scholastic variables were systematically related to HD-baseline with higher odds for HD in children who reported lower GPA (2.45, (1.70–3.53)), those who were more absent from school (1.99 (1.40–2.80)), and those who had more unexcused school absences (1.75 (1.17–2.60)). Those children who were currently involved in team sports (2.35 (1.12–4.94)), those who quit team sports (1.78 (1.04–3.06)), and those who reported longer involvement in sports (1.38 (1.10–1.73)), were more likely to be engaged in HD (Table 4). 

Grade point average was associated with HD-follow-up (2.65 (1.61–4.37)), as were school absences (1.58 (1.09–2.26)) and unexcused school absences (1.49 (1.06–2.11)), with higher odds for HD among those who performed poorly in school. There was no significant association between sport factors and HD-follow-up (Table 4).

Higher odds for HD-initiation were found for adolescents who quit an individual sport (13.13 (2.67–64.62)), who reported longer involvement in sport (2.10 (1.18–3.72)), and who achieved better competitive results in sport (3.15 (1.19–8.34)) (Table 4).

## 4. Discussion

There are several important findings in this study. First, scholastic variables were systematically related to HD in both testing waves (i.e., the beginning of the 3rd year of high school and the end of high school education), but scholastic variables were not found to be a predictor of HD-initiation. Second, sport participation was recognized as a risk factor with higher odds of HD-baseline. Additionally, baseline sport factors were directly related to HD-initiation, with higher odds for alcohol drinking initiation in those adolescents who achieved better sport results and were longer involved in sport. First, we will shortly overview data on prevalence of HD in studied adolescents

### 4.1. Prevalence of Harmful Drinking

With approximately 30% of adolescents reporting HD, the prevalence of alcohol drinking in Croatian adolescents remains alarmingly high [38]. These figures are almost identical to a previous study that was done in 2010 in Split, one of the cities also included in this study [32]. However, while the previously cited study investigated adolescents at one time-point (i.e., the end of the high school), this study expands previous knowledge by evidencing a certain increase in HD between 16 and 18 years of age of approximately 6%. When explaining such a high prevalence of HD, several very important issues deserve attention. First, alcohol drinking is a socially acceptable behavior in the whole territory of former Yugoslavia, including Croatia [22,32]. This is largely influenced by the fact that Croatia is a Mediterranean country, where the Mediterranean style of drinking is common (i.e., alcohol consumption is regular during meals, but intoxication is not socially acceptable), and drinking is associated with religious affiliation (i.e., Croatians are mostly Roman Catholics, which means that there are no religious boundaries against alcohol consumption). Another contributing factor is a long tradition of wine growing and winemaking, especially in the Adriatic region of the country [39]. Consequently, adolescents start to consume alcohol at early ages, although this occurs mostly in a familial environment. This kind of alcohol consumption is often considered as “controlled” and, therefore, not harmful. 

Meanwhile, regardless of “controllability of drinking” in situations where alcohol is consumed together with responsible adults and family, the early initiation of alcohol drinking is known to be a risk factor for HD in a “non-controlled” environment, such as gatherings in bars and clubs, where alcohol purchasing and consumption is neither controlled nor supervised [40]. In the territories studied in our research, the problem emerges exponentially during the summer and tourist season (see Figure 1; both regions included in this study are located on the Adriatic Sea), when open-air summer parties are the most popular types of social gatherings. In such circumstances there is practically no control over purchasing alcohol, which almost certainly aggravates the problem of HD among adolescents. Regardless of the background of such high prevalence, the HD figures are in accordance with those reported for other countries in the region, such as Kosovo and Bosnia-and-Herzegovina (approximately 30% of HD prevalence), which clearly indicate that alcohol drinking is an emerging problem for the entire territory of former Yugoslavia, regardless of ethnicity and/or religious affiliation [23,29,36].

### 4.2. Sport Factors and Harmful Drinking 

Our results indicated higher risk for HD at study baseline among those adolescents who were actively involved in team sports and among those who quit team sports, relative to those who were never involved in team sports. Although it must be noted that the group of those “non-involved” also included participants who participated in individual sports, it seems that participation in team sports should be considered as a risk factor for HD at the age of 16 years. Additionally, since results showed no association between team-sport participation and HD follow-up, it seems reasonable to conclude that team-sport athletes start to consume alcohol at harmful levels earlier than (i) their non-athletic peers and (ii) those involved in individual sports. In explaining such findings, the problem of age-segregation (age-bonding) is interesting, especially since previous studies reported this issue as being potentially related to substance misuse in adolescence [27]. 

Social networking with older adolescents increases the risk for substance misuse, including alcohol consumption [41]. Sport competitions and training are mostly organized for certain age groups. Therefore, it is considered that age-bonding in sports decreases the possibility of “negative social influence” of older peers [27]. As a result, participation in sport during adolescence may protect young people of being involved in HD as well. However, with respect of team-sports, this is less likely because team sport training involves a larger group of athletes (i.e., there is no possibility of individual training). Consequently, in a majority of team-sports, different age-groups are often clustered, simply because this is an easier organization for training. As a result, social networking with older peers is rather more prevalent in team-athletes than in their non-athletic peers. In contrast, participation in individual sports allows “individual training”. Individual-sport athletes are, therefore, less likely to make a social network with their older peers, and in such circumstances, the previously explained protective effect of age-bonding against HD is more probable [27]. The non-significant association between participation in individual sports and HD-baseline at least partially supports this statement. 

Although previous discussion is based on the comparison of associations established for baseline- and follow-up measurement, previous cross-sectional studies confirm our conclusions [25,36]. In short, evidence suggests that athletes typically consume alcohol in social environments, which leads to more consumption given the inherent social nature of team sports. In such cases, post-exercise drinking is rationalized and justified by athletes in many ways, including ‘‘everyone is doing it,’’ ‘‘I only drink once a week,’’ and ‘‘I can run/sauna it off the next morning.’’ In some cases, these episodes are romanticized, and the drinking prowess of the athletes is admired. The media covers such ‘‘incidents’’ extensively; consequently, adolescent team-sport athletes do not recognize drinking as harmful [25]. The finding on association between quitting team-sports and the prevalence of HD at baseline will be discussed shortly. 

While quitting team-sports was evidenced as a risk factor for HD-baseline, quitting individual sports increased the likelihood of HD-initiation until follow-up. These results confirm previous considerations on that problem [23], as well as our initial experimental approach, where involvement in individual- and team-sports were observed separately. Evidently, the particular circumstances, unique socio-cultural background, and psycho-physiological requirements of individual- and team-sport-participation results in specific associations between these factors and HD in adolescence, as already suggested [25]. Therefore, team-sport participation results in initiation of HD before the age of 16 years, while adolescents who quit individual sport are more likely to initiate HD from 16 to 18 years. Meanwhile, there is no evidence that active participation in individual sports is a risk factor for HD in the studied period. 

It has already been hypothesized that the discontinuation of involvement in sport should be considered a risk factor for HD [23], and for that reason, this hypothesis is specifically investigated herein. With regard to empirical evidence, we have not been able to find prospective study that examined this problem, but our results agree with previous cross-sectional studies [25,42]. For example, active participation in individual sports was protective against alcohol consumption in 18-year-old boys [25], while discontinuation from individual sports was a factor of increased likelihood of alcohol drinking in 17-year-old girls [42]. On the other hand, team-sport participation is recognized as a risk factor for alcohol consumption in rugby athletes (i.e., typical team-sport), which is explained by specific socio-cultural circumstances, including frequent post-sport gatherings where alcohol consumption is almost mandatory [43]. 

Because of the cross-sectional nature of the previous investigations, the causality between sport-factors and alcohol drinking was unknown, but in this study, we have clearly depicted that discontinuation in individual sport involvement precedes the initiation of HD in this period of life. However, longer involvement in sport and better sport achievement (sport result) are also predictive for HD-initiation. Both of these variables actually highlight commitment to sport. Evidently, those who achieved sport results and who participated in sport for a long time are at specific risk for HD-initiation between 16 and 18 years of age. Therefore, even adolescents who are most committed to sport and achieved excellent competitive results are vulnerable to HD in this period. 

The fact that HD occurs even in the most successful and most committed athletes is not in agreement with theories where sport is considered as protective against substance misuse in adolescence [27]. For example, it is theorized that sport participation takes time, and then the time is spent on training and competition, therefore leaving less time available for other social activities where alcohol drinking is more likely [27]. Additionally, sport is organized and supervised by adult coaches, and their monitoring should limit problem behavior in children who practice sports. Finally, even an orientation toward success, which in sport is directly influenced by level of physical capacities (i.e., sport result greatly depends on one’s physical performance), should act as protective factors against HD simply because alcohol intoxication directly jeopardizes the ability to train and compete [27]. Despite all these stated hypotheses of the protective effects of sport, the adolescent athletes included in our study were found as having a higher risk of HD than their non-athletic peers. 

Although this may seem surprising at first glance, our finding on sport participation during adolescence as predictive of HD is actually in accordance with studies that examined the problem prospectively in other world regions, including Norway [27] and the USA [44,45]. Knowing the situation in Croatia, the authors share the opinion that the overall acceptance of alcohol in the sport community is the main explanation for the high occurrence of HD in young athletes. Further, we must agree with some previous considerations where the competitive nature of the sport is highlighted as a possible explanation for the high occurrence of alcohol consumption in adolescent athletes (i.e., a “competitive climate” encourages young athletes to show that they “can hold the liquor”) [46]. Finally, it must not be ignored that sport participation may be very stressful [47]. The high levels of stress are particularly evident in the period of adolescence studied, when most rigid sport selection occurs (i.e., selection between junior- and senior-age levels). Alcohol may be introduced as a coping mechanism for sport-related anxiety, which explains the findings regarding the influence of sport-result and sport-experience on HD-initiation. 

### 4.3. Scholastic Variables and Harmful Drinking 

Results indicated strong cross-sectional associations between scholastic achievement and HD at baseline and at follow-up with lower scholastic performance in children who reported HD. These results are in agreement with previous studies that examined the problem globally, as well as results of investigations that sampled adolescents from southeastern Europe [25,29]. Meanwhile, to the best of our knowledge, this is one of the first studies where adolescents from Croatia were systematically observed and probably the first one where adolescents were observed across two time points. Therefore, we may say that our results advance the knowledge by informing us, basically, about an unchanged association between scholastic achievement and HD in the studied period. The poorer scholastic achievement in children who consume alcohol is explainable by (at least) three mechanisms. 

The first mechanism of association is essentially physiological, where alcohol consumption is proposed as a “cause” of low scholastic achievement [48]. In short, studies on adolescent brain development suggest that early heavy alcohol use may have negative effects on the physical development of the brain structure, which, consequently, can result in the deterioration of learning capacities and finally to poor grades [49,50]. The main criticism of such explanations comes from the understandable interpretations about the relatively short period of alcohol consumption in adolescents (and the consequent small possibility of a negative impact of alcohol on brain structures). Additionally, our results on the negative association between other scholastic variables (i.e., behavioral grade, absence, unexcused absence) with HD, point to another possible criticism of such a physiologically based explanation of the negative associations between alcohol drinking and scholastic result. Basically, even if poor GPAs are a result of the negative effect of alcohol on learning, this effect is hardly acceptable for other studied measures of scholastic achievement, which clearly do not depend on learning capacity. 

The second, mainly socio-psychologically based, explanation is also probable. In short, students who perform poorly in school are often in non-controlled, “out of school situations”, and this is supported in studies where authors reported strong correlations between absence from school and alcohol consumption in adolescents [51]. As a result, alcohol consumption is more prevalent in those who do not do well in school, but this time, alcohol drinking is a result, and low scholastic achievement is the cause. The third explanation is based on the theory of problem behavior [52]. Some individuals have a tendency to nonconventionality and, to some extent, a tendency toward “problem behavior”. For those persons, alcohol drinking may appear in tandem with low scholastic achievement simply because problematic behaviors appear “in tandem”. If we accept this explanation, there is no causal relationship between alcohol drinking and scholastic failure, but both appear as a result of specific socio-psychological characteristics of certain people. Of course, this explanation may be accounted for some adolescents, but it hardly accounts for all of them who reported alcohol consumption and scholastic failure. 

This study was partially based on a lack of knowledge about the causality between scholastic failure and alcohol drinking in adolescence. Therefore, we aimed to prospectively investigate this problem and to possibly define the predictors of HD initiation. However, scholastic variables observed at study baseline were not identified as significant predictors of HD-initiation. Therefore, we may say that, for this age group, scholastic failure cannot be observed as a predictor of HD. There is a certain possibility that associations of such kind should be identified at an earlier age (i.e., the first two years of high school), and, therefore, future studies are warranted. 

### 4.4. Study Limitations and Strengths

The data were self-reported; therefore, adolescents may lean toward socially acceptable answers. However, we believe that the study design and strict preservation of participant anonymity decreased this possibility. Additionally, we must note that alcohol drinking in Croatia is socially acceptable behavior, that we studied older adolescents, and that the majority of adolescents involved in the study initiated HD earlier in life (before the age of 16 years). Therefore, the generalizability of the results is possible to similar regions and samples of participants. Also, study lacks information about age of drinking onset. This is an important problem since drinking onset may correlate with initiation of sports-participation. Finally, this study lacked important information on recreational sport participation (i.e., self-exercising in fitness centers, running/jogging, outdoor activities), which could be important determinants of the prevalence of alcohol consumption. 

This is likely one of the first investigations where sport-factors and scholastic-factors were prospectively observed as potential predictors of HD in adolescence. The controlled study design, specific population with a high prevalence of HD, and relatively high retention rate (i.e., small drop-out rate) are important strengths of the study. Therefore, we believe that the results, although not the final word on this problem, will contribute to overall knowledge. 

## 5. Conclusions 

The prevalence of alcohol drinking, including HD, in Croatian adolescents remains high. While this study included older adolescents from one specific region of the country, future studies should explore the problem in other regions and among younger adolescents. While clear reasons for such disturbing figures on HD prevalence remain relatively unknown, early initiation of alcohol consumption (i.e., most of the studied adolescents began to drink alcohol before the age of 16), social acceptance of alcohol drinking, low prices for liquor, and nonsystematic control of alcohol purchasing for minors are among the most likely causes of the high prevalence of HD. 

Sport factors were specifically associated with HD, with discontinuation in sport participation as the factor of increased risk for HD in cross-sectional analyses. The results revealed an important finding on increased risk for HD initiation during the study course for those adolescents who reported high sport-commitment (i.e., those who achieved sport result and were involved in sport for a longer time). This finding should be explained bearing in mind forthcoming sport-selection between junior and senior-age level (i.e., transition from amateur to professional sport) and the associated stress placed on young athletes. Therefore, sport authorities should be informed of these findings in order to pay special attention to young athletes in this period of life and to make additional efforts to reduce the possibility of HD occurrence in sport settings (i.e., post-sport social gatherings and celebrations). 

Scholastic variables were related to HD in cross-sectional analyses, with a higher likelihood of HD in those adolescents who reported lower school success. However, scholastic variables evidenced at baseline were not significantly related to HD initiation between 16 and 18 years of age. This points to two possible conclusions. First, it is possible that alcohol drinking should be seen as a predictor of poor scholastic achievement. Second, it is possible that both alcohol drinking and scholastic failure are actually results of problematic behavior in adolescence. For a more profound analysis, studies in children of a younger age are warranted. 

## Figures and Tables

**Table 1:** Scholastic variables at baseline and follow-up, and differences on a basis of harmful drinking —HD (MW—Mann Whitney Z values).

Scholastic Variables	Baseline			Follow-Up		
	Non-HD	HD	MW	Non-HD	HD	MW
	F (%)	F (%)	Z (p)	F (%)	F (%)	Z (p)
**Grade point average**						
Excellent	84 (16.8)	16 (11.11)	−3.26 (0.01)	96 (20.87)	30 (16.3)	−1.27 (0.2)
Very good	256 (51.2)	56 (38.89)		222 (48.26)	88 (47.83)	
Average	122 (24.4)	68 (47.22)		122 (26.52)	64 (34.78)	
Under average	18 (3.6)	4 (2.78)		16 (3.48)	2 (1.09)	
Poor	18 (3.6)	0 (0)		4 (0.87)	0 (0)	
Missing	2 (100)	0 (0)		0 (0)	0 (0)	
**Absences from school**						
Less than 10 hours	154 (30.8)	32 (22.22)	−2.72 (0.01)	90 (19.57)	22 (11.96)	−4.56 (0.01)
10-20 hours	210 (42)	60 (41.67)		174 (37.83)	52 (28.26)	
21-40 hours	122 (24.4)	38 (26.39)		160 (34.78)	74 (40.22)	
More than 40 hours	14 (2.8)	14 (9.72)		36 (7.83)	36 (19.57)	
Missing	0 (0)	0 (0)		0 (0)	0 (0)	
**Unexcused absences**						
None	318 (63.6)	60 (41.67)	−3.65 (0.01)	244 (53.04)	64 (34.78)	−4.49 (0.01)
1-5 hours	100 (20)	54 (37.5)		106 (23.04)	56 (30.43)	
6-10 hours	36 (7.2)	22 (15.28)		50 (10.87)	16 (8.7)	
11-20 hours	24 (4.8)	6 (4.17)		26 (5.65)	14 (7.61)	
More than 20 hours	22 (4.4)	0 (0)		34 (7.39)	34 (18.48)	
Missing	0 (0)	2 (1.39)		0 (0)	0 (0)	
**Behavioral grade**						
Exceptional	430 (86)	128 (88.89)	0.97 (0.32)	388 (84.35)	136 (73.91)	−3.14 (0.01)
Average	58 (11.6)	16 (11.11)		68 (14.78)	42 (22.83)	
Poor	12 (2.4)	0 (0)		4 (0.87)	6 (3.26)	
Missing	0 (0)	0 (0)		0 (0)	0 (0)	

**Table 2:** Sport factors at baseline and follow-up, and differences on a basis of harmful drinking—HD (MW—Mann Whitney Z values; Chi square test).

Sport Factors	Baseline			Follow-Up		
	Non-HD	HD	MW	Non-HD	HD	MW
	F (%)	F (%)	Z (p)	F (%)	F (%)	Z (p)
**Sport success/result**						
Never competed	190 (38)	34 (23.61)	2.8 (0.06)	162 (35.22)	64 (34.78)	0.08 (0.93)
Local rank	210 (42)	74 (51.39)		204 (44.35)	84 (45.65)	
National rank	84 (16.8)	36 (25)		74 (16.09)	30 (16.3)	
International rank	14 (2.8)	0 (0)		20 (4.35)	6 (3.26)	
Missing	2 (0.4)	0 (0)		0 (0)	0 (0)	
**Socioeconomic status**						
Under average	14 (2.8)	4 (2.78)	0.26 (0.79)	8 (1.74)	8 (4.35)	2.25 (0.02)
Average	424 (84.8)	124 (86.11)		390 (84.78)	160 (86.96)	
Above average	60 (12)	16 (11.11)		62 (13.48)	16 (8.7)	
Missing	2 (0.4)	0 (0)		0 (0)	0 (0)	
**Experience in sport**						
Never been involved	72 (14.4)	12 (8.33)	−0.91 (0.36)	96 (20.87)	40 (21.74)	−0.91 (0.35)
<1 year	36 (7.2)	20 (13.89)		26 (5.65)	10 (5.43)	
2–5 years	172 (34.4)	42 (29.17)		138 (30)	42 (22.83)	
>5 years	220 (44)	70 (48.61)		200 (43.48)	92 (50)	
Missing	0 (0)	0 (0)		0 (0)	0 (0)	
			**Chi Square (p)**			**Chi Square (p)**
**Individual sport participation**						
Yes, participating	104 (20.8)	22 (15.28)	4.23 (0.12)	78 (16.96)	16 (8.7)	8.10 (0.02)
Quit	218 (43.6)	76 (52.78)		230 (50)	94 (51.09)	
Never	178 (35.6)	46 (31.94)		152 (33.04)	74 (40.22)	
Missing	0 (0)	0 (0)		0 (0)	0 (0)	
**Team sport participation**						
Yes, participating	98 (19.6)	48 (33.33)	20.88 (0.01)	82 (17.83)	28 (15.22)	9.18 (0.01)
Quit	250 (50)	76 (52.78)		220 (47.83)	84 (45.65)	
Never	152 (30.4)	20 (13.89)		158 (34.35)	72 (39.13)	
Missing	0 (0)	0 (0)		0 (0)	0 (0)	

**Table 3:** Socioeconomic status, parental conflict and gender (covariates) at baseline and follow-up, and differences on a basis of harmful drinking—HD (MW—Mann Whitney Z values).

Covariates	Baseline			Follow-Up		
	Non-HD	HD	MW	Non-HD	HD	MW
	F (%)	F (%)	Z (p)	F (%)	F (%)	Z (p)
**Socioeconomic status**						
Under average	14 (2.8)	4 (2.78)	0.26 (0.79)	8 (1.74)	8 (4.35)	2.25 (0.02)
Average	424 (84.8)	124 (86.11)		390 (84.78)	160 (86.96)	
Above average	60 (12)	16 (11.11)		62 (13.48)	16 (8.7)	
Missing	2 (0.4)	0 (0)		0 (0)	0 (0)	
**Conflict with parents**						
Almost never	80 (16)	22 (15.28)	−4.17 (0.01)	110 (23.91)	52 (28.26)	−0.55 (0.57)
Rarely	230 (46)	36 (25)		202 (43.91)	62 (33.7)	
Occasionally	164 (32.8)	64 (44.44)		132 (28.7)	56 (30.43)	
Frequently	26 (5.2)	22 (15.28)		16 (3.48)	14 (7.61)	
Missing	0 (0)	0 (0)		0 (0)	0 (0)	
**Gender**						
Male	250 (50)	96 (66.66)	12.49 (0.01)	222 (48.26)	124 (67.39)	19.34 (0.01)
Female	250 (50)	48 (33.33)		238 (51.74)	60 (32.61)	
Missing	0 (0)	0 (0)		0 (0)	0 (0)	

**Table 4:** Odds ratio (OR) with 95% Confidence Intervals (CI) for harmful drinking (HD) at baseline and follow-up, and initiation of HD during the study course.

Predictors	Baseline		Follow-Up		Initiation ^¥^	
	Crude OR (95% CI)	Adjusted OR (95% CI)	Crude OR (95% CI)	Adjusted OR (95% CI)	Crude OR (95% CI)	Adjusted OR (95% CI)
**Grade point average ^cont^**	2.76 (1.94–3.94)	2.45 (1.70–3.53)	2.92 (1.82–4.65)	2.65 (1.61–4.37)	3.05 (1.25–7.49)	2.99 (0.94–9.50)
**Absences ^cont^**	1.98 (1.43–2.75)	1.99 (1.40.2.80)	1.45 (1.02–2.05)	1.58 (1.09–2.26)	1.36 (0.68–2.72)	1.14 (0.65–3.22)
**Unexcused absences ^cont^**	2.16 (1.44–3.23)	1.75 (1.17–2.60)	1.63 (1.15–2.28)	1.49 (1.06–2.11)	1.35 (0.54–3.36)	1.22 (0.44–3.37)
**Behavioral grade ^cont^**	3.00 (1.12–8.01)	1.9 (0.7–5.2)	/	/	/	/
**Individual sport**						
Yes, participating	1.50 (0.71–2.96)	1.39 (0.71–2.76)	1.42 (0.45–3.66)	1.06 (0.39–2.85)	/	/
Quit	1.81 (1.07–3.09)	1.57 (0.91–2.72)	1.65 (0.85–3.19)	1.47 (0.75–2.90)	6.00 (1.73–20.81)	13.13 (2.67–64.62)
Never	REF	REF	REF	REF	REF	REF
**Team sport**						
Yes, participating	2.55 (1.26–5.16)	2.35 (1.12–4.94)	0.95 (0.43–2.11)	0.57 (0.34–1.37)	2.27 (0.42–12.34)	1.50 (0.21–10.61)
Quit	1.96 (1.16–3.31)	1.78 (1.04–3.06)	1.97 (0.97–3.99)	1.59 (0.77–3.27)	1.09 (0.38–3.12)	0.82 (0.23–2.93)
Never	REF	REF	REF	REF	REF	REF
**Sport success/result ^cont^**	1.38 (0.99–1.90)	1.24 (0.89–1.74)	1.32 (1.04–1.69)	1.18 (0.91–1.53)	3.67 (1.48–9.11)	3.15 (1.19–8.34)
**Experience in sport ^cont^**	1.45 (1.18–1.80)	1.38 (1.10–1.73)	1.75 (1.13–2.73)	1.49 (0.95–2.32)	1.75 (1.12–2.73)	2.10 (1.18–3.72)

Notes: REF—referent value, Crude—nonadjusted logistic regression, Adjusted—logistic regression adjusted for age, gender, socioeconomic status and conflict with parents, ^cont^ — denotes variables observed as continuous for the purpose of logistic regression calculations, /—logistic regression was not calculated due to “null” frequencies, ^¥^ —participants who were identified as being HD at study baseline were not included in this analysis.
